# The seeding of climate smart health care

**DOI:** 10.2471/BLT.21.020221

**Published:** 2021-02-01

## Abstract

After a slow start, climate-smart health initiatives are gaining momentum. Gary Humphreys and Sophie Cousins report.

Alex Williams remembers the storm well. It hit the Caribbean island of St Vincent on Christmas Eve 2013, delivering torrential rain and high winds. “Communities lost electricity and water and 39 health-care clinics were shut down along with the island’s only referral hospital,” Williams says. “The only hospital to withstand the shock was Georgetown.”

Georgetown Hospital’s resilience was the result of a retrofit undertaken as part of a Safe Hospitals Initiative pilot project that was implemented with funding from the United Kingdom of Great Britain and Northern Ireland’s Department for International Development and the support of the Pan American Health Organization (PAHO) in two Caribbean locations in 2012.

An electrical engineer and technical consultant with PAHO, Williams worked on the Georgetown Hospital retrofit and is proud of what was achieved there. “The hospital’s roof was repaired and solar panels were installed, and the plumbing and energy systems were renovated,” he explains. “When the storm hit, the hospital not only continued to function, it became a vital source of fresh water to the surrounding community in the following weeks.”

It is generally recognized that the Caribbean states are among the earliest adopters of health system resilience strategies, an endeavour undertaken with strong support from PAHO. They are also credited for their efforts to help reduce the environmental impact of health systems.

“We know that natural hazards, including climate-change-related events such as intense storms, can have a major impact on health systems, but it is important to recognize that health systems also impact the environment, and, by extension, climate change,” says senior advisor for disaster preparedness and response at PAHO, Dr Dana Van Alphen.

“To really meet the challenges faced, we need to reach beyond individual health facilities, and cover the full range of health system functions.”

Diarmid Campbell-Lendrum

To address these interrelated challenges, Van Alphen proposed combining disaster resilience and greening measures in an approach that has come to be referred to as climate smart health care. After supporting implementation of the Safe Hospitals Initiative pilot, Van Alphen led an effort to identify facilities in Caribbean states and territories that had climate smart potential. In the end, 54 hospitals and health centres in seven Caribbean countries were chosen for upgrades in an initiative that is targeted for completion in 2022.

While such initiatives are clearly laudable, some experts question whether they are enough to achieve the progress required on health system resilience or health system greening to meet the relevant goals and targets set out in the sustainable development goals or United Nations Framework Convention on Climate Change (UNFCCC).

“To really meet the challenges faced, we need to reach beyond individual health facilities, and cover the full range of health system functions, including medical product development, supply chain design and management, and health information systems,” says Diarmid Campbell-Lendrum, head of the World Health Organization’s (WHO) climate change unit. “We also need to go beyond the formal health sector, and address the determinants of health, such as clean and reliable energy, water and sanitation services, something that will require a whole-of-government approach.”

According to Campbell-Lendrum, to date governments have been slow to embrace climate-smart health agendas, despite recent progress at country level and the commitments made as part of the Paris Agreement on climate change, which was signed in 2015 within the UNFCCC, and includes agreements on greenhouse-gas-emissions mitigation and funding.

Campbell-Lendrum highlights the lack of funding support for climate smart health as a major obstacle to progress, pointing out that less than 1% of international climate finance has been allocated to support health projects. Notable in this regard is the Green Climate Fund (GCF), which was set up as the main financial instrument for the Paris Agreement and was designed to transfer public capital from developed countries to developing countries to support their transition to lower-carbon and more climate resilient societies. Although the GCF Board has identified health as a priority action area, it has yet to fund a single health project.

Despite this historical lack of momentum, Campbell-Lendrum believes that combining resilience and greening strategies – and weaving both into universal-health-coverage-related reforms – has the potential to increase government interest in and support for urgently needed change, particularly governments in developing countries.

“Connecting environmental sustainability, climate resilience and universal health coverage together is much more compelling than simply asking governments in developing countries to cut their carbon emissions – as they have made very little historical contribution to global warming,” he says.

Responding to growing demand from countries, WHO published guidance titled *Climate resilient and environmentally sustainable health care facilities* in late 2020 and is incorporating it into its growing portfolio of climate change and health projects around the world.

Josh Karliner, international director, Program and Strategy at Health Care Without Harm (HCWH), a nongovernmental organization that works on health system greening issues, takes a similar view of recent history, but he is more optimistic about the prospects for project-driven change, citing the example of HCWH’s work on mercury waste.

“HCWH has worked for more than 15 years on substituting mercury-based medical devices with accurate, affordable alternatives and that work started in a single hospital in Boston,” Karliner says.

Working in partnership with WHO, HCWH then seeded the project in hospitals worldwide, demonstrating that medical devices using alternatives to mercury, including thermometers and blood pressure devices, were both accurate and affordable. The initiative has gone on to become a worldwide movement with goals now enshrined in a global treaty known as the Minamata Convention, which called for the phasing out of mercury-based medical products in 2020.

HCWH has seen similar momentum developing with alternative energy initiatives in both resource-rich and -poor health-care settings. A notable example is in Chhattisgarh in central India where the Chhattisgarh State Renewable Energy Development Agency installed solar panels on the rooftops of medical facilities, ensuring round-the-clock power supply.

“We expect to have health systems committing to zero emissions by 2050 on every continent.”

Josh Karliner

“They started with just a few hospitals implementing renewable energy, then scaled to 900 health centres,” Karliner says. “They just now committed to 100% renewable energy for the entire health system in the state which is one of the poorest in India.”

According to Karliner, the key to HCWH’s success is the steady bottom-up development of a network of health authorities which the organization has cultivated over the past decade.

That network got a boost in 2015 when, at the Paris Climate Conference, the HCWH launched the Health Care Climate Challenge, an initiative designed to encourage health-care institutions around the world to play a leadership role in addressing climate change.

The initiative now has more than 300 participating institutions representing the interests of about 22 000 hospitals and health centres, all committed to working on and reporting on resilience mitigation and leadership actions.

Karliner is keen to point out that the Challenge is also beginning to draw in national government agencies including Ethiopia’s Ministry of Health and the United Kingdom’s National Health Service, which has been working on climate-friendly policy for some time, and in September 2020 announced a net zero direct emissions target for 2040, becoming the****world’s first national health system to do so.

“The National Health Service committing to net zero is indicative of the growing momentum on decarbonization and resilience among health-care systems,” Karliner says, pointing to the inclusion of a health component in the UNFCCC’s Race to Zero campaign as another positive indicator.

The Race to Zero campaign aims to bring together net zero commitments from cities, businesses and investors across the climate action community in the run up to the 2021 UNFCCC Climate Change Conference, also known as COP26, which is scheduled to be held in Glasgow, Scotland in November 2021.

HCWH was named as an official Race to Zero partner in November of last year and is putting together a cohort of health institutions that will participate in the race seeking to meet targets set on emissions and align the global health sector with the ambition of the Paris Agreement.

“We will announce the cohort in the first half of 2021 – and will be adding to that cohort as we build towards COP26,” Karliner says. “By that time, we expect to have health systems committing to zero emissions by 2050 on every continent.”

Campbell-Lendrum also senses a change in the weather. “As climate issues become more central to the core concerns of national governments and as low-carbon technologies become more affordable, we are moving towards a tipping point on climate smart health,” he says.

“Ultimately, we will have won this battle when we can move beyond projects and initiatives and reach a point where building climate resilient and environmentally sustainable health facilities and systems is so normal we don’t even need to talk about it.”

**Figure:**
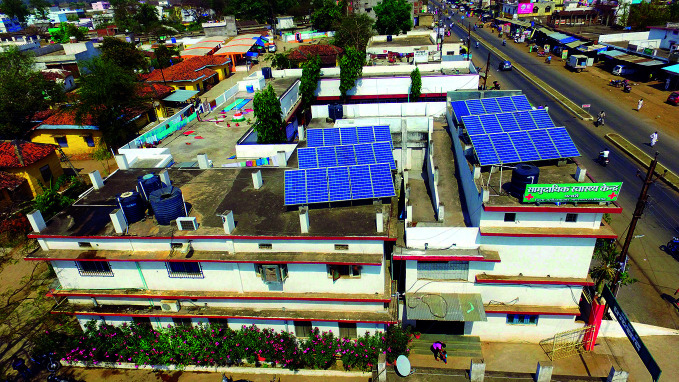
A community health centre powered by solar panels in the state of Chhattisgarh, India.

CREDA / Shri Sanjeev Jain

**Figure:**
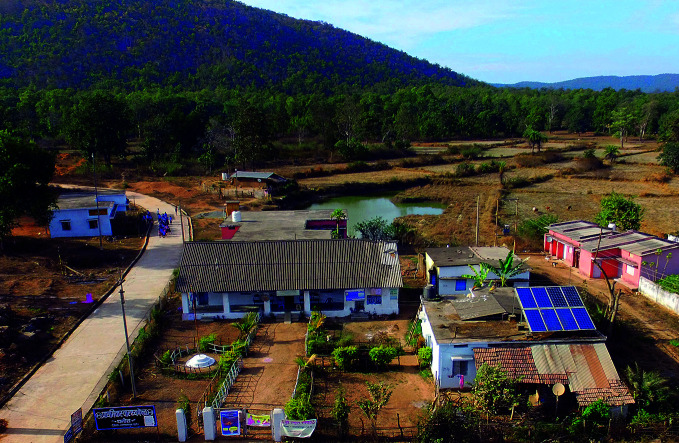
A primary health centre powered by solar panels in the state of Chhattisgarh, India.

CREDA / Shri Sanjeev Jain

